# UAV-PDD2023: A benchmark dataset for pavement distress detection based on UAV images

**DOI:** 10.1016/j.dib.2023.109692

**Published:** 2023-10-15

**Authors:** Haohui Yan, Junfei Zhang

**Affiliations:** a School of Artificial Intelligence, Hebei University of Technology, Tianjin 300401, China; b School of Civil and Transportation Engineering, Hebei University of Technology, Tianjin 300401, China

**Keywords:** Pavement distress detection, UAV, Computer vision, Deep learning

## Abstract

The UAV-PDD2023 dataset consists of pavement distress images captured by unmanned aerial vehicles (UAVs) in China with more than 11,150 instances under two different weather conditions and across varying levels of construction quality. The roads in the dataset consist of highways, provincial roads, and county roads constructed under different requirements. It contains six typical types of pavement distress instances, including longitudinal cracks, transverse cracks, oblique cracks, alligator cracks, patching, and potholes. The dataset can be used to train deep learning models for automatically detecting and classifying pavement distresses using UAV images. In addition, the dataset can be used as a benchmark to evaluate the performance of different algorithms for solving tasks such as object detection, image classification, etc. The UAV-PDD2023 dataset can be downloaded for free at the URL in this paper.

Specifications Table

**Table:** 

Subject	Computer Vision and Pattern Recognition, Computer Science Applications, Pavement Engineering
Specific subject area	UAV-based Pavement Distress Detection, Object Detection and Classification
Type of data	2D-RGB Images (.jpg), Annotation Files (.xml) in Pascal VOC Format
How data were acquired	UAV payload gimbal camera: DJI Zenmuse H20, 1/1.7" CMOS, 20 million effective pixels; DFOV: 66.6°-4° focal length. Pavement images (.jpg) were collected using a UAV flying at less than 1 m/s at 30 m above the ground. The *labelme* tool was used to create XML files to annotate the pavement distress present in the images
Description of data collection	The pavement distress images were collected during the day. Some images were acquired 1 h after rain, and a wide variety of weather and lighting conditions were taken into account when capturing the images. The RGB images were captured by adjusting the camera angle of the UAV to -90°, vertically with the field. The UAV utilized two different shooting methods, hovering and slow movement, to provide clear views of distress images. The maximum shooting width was up to 15 m (the width of four lanes).
Data source location	Country: China, City: Tianjin
Data accessibility	Repository Name:UAV-PDD2023 Direct URL: https://zenodo.org/record/8429208 DOI:10.5281/zenodo.8429208

## 1 Value of the Data

• The UAV-PDD2023 dataset, captured by UAVs, provides a basis for pavement distress detection using deep learning. It is highly useful for municipal authorities and pavement agencies to conduct low-cost pavement condition monitoring.
• The UAV-PDD2023 dataset is of great value for developing new deep convolutional neural network architectures or modifying existing architectures to enhance network performance. Researchers can utilize this data for algorithm training, validation, and testing, aiming to develop algorithms for pavement distress detection using UAV.
• The dataset supports detection and classification of pavement cracks Longitudinal cracks (LC), Transverse cracks (TC), Alligator cracks (AC), Oblique crack(OC), Repair(RP) and Potholes (PH). It can be further expanded to include other distress categories.
• Researchers can utilize these datasets to benchmark the performance of various algorithms for addressing similar problems, such as image classification and object detection.

## 2 Data Description

The UAV-PDD2023 image dataset consists of 2440 images collected from China, with over 11,158 instances of pavement distresses. Pavement images were captured using a UAV. The effectiveness of UAVs in the health monitoring of civil infrastructure has been demonstrated [1,2]. To enhance the practicality of the dataset, it incorporates images of pavement distress captured during clear weather conditions as well as within an hour after rainfall. Additionally, the dataset encompasses images taken from roads with varying construction qualities. The roads in the dataset consist of highways, provincial roads, and county roads. This dataset includes annotations for six categories of distresses: Longitudinal cracks (LC), Transverse cracks (TC), Alligator cracks (AC), Oblique crack (OC), Repair (RP) and Potholes (PH).

**Fig. 1:**
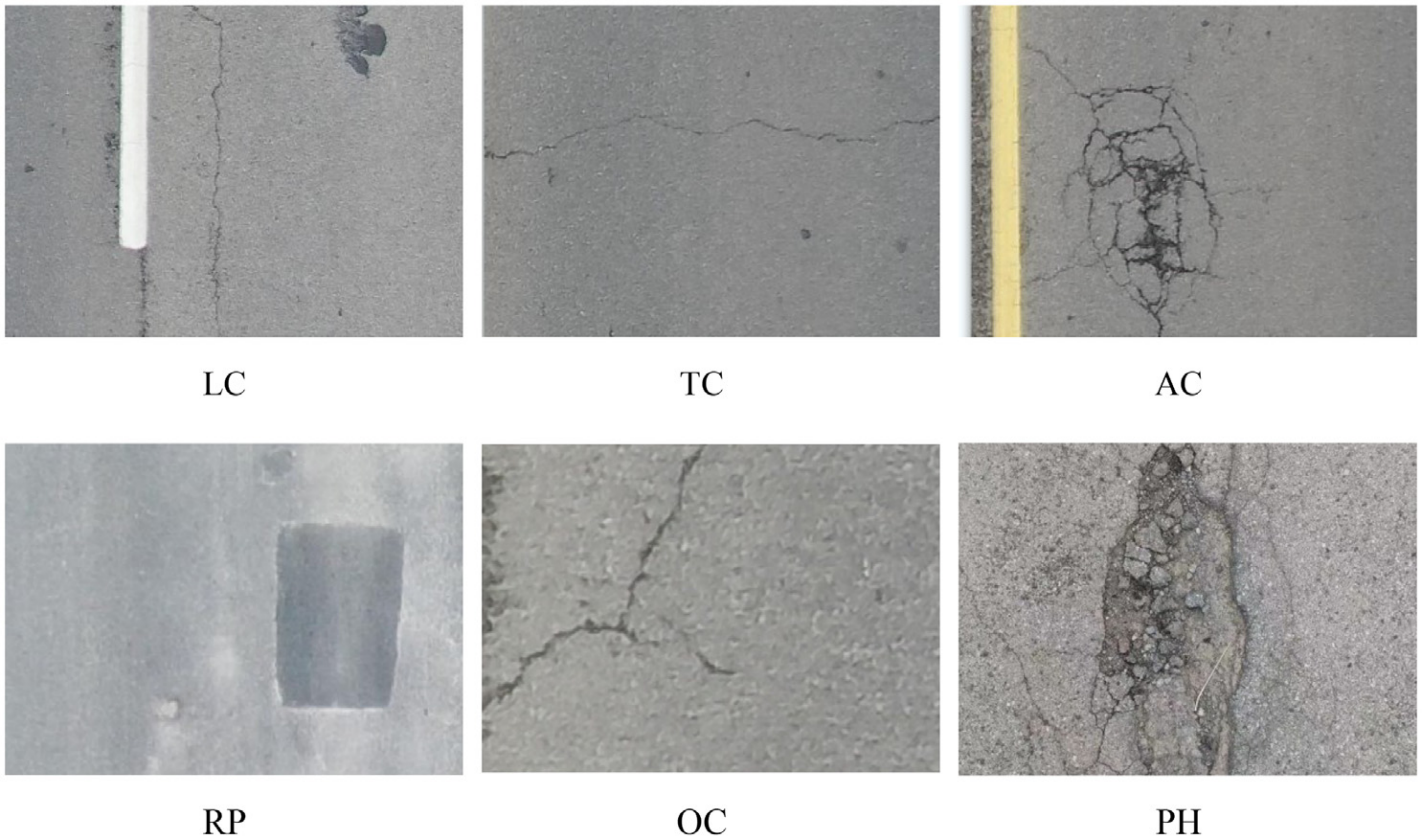
Sample Images of pavement distresses.

**Fig. 2:**
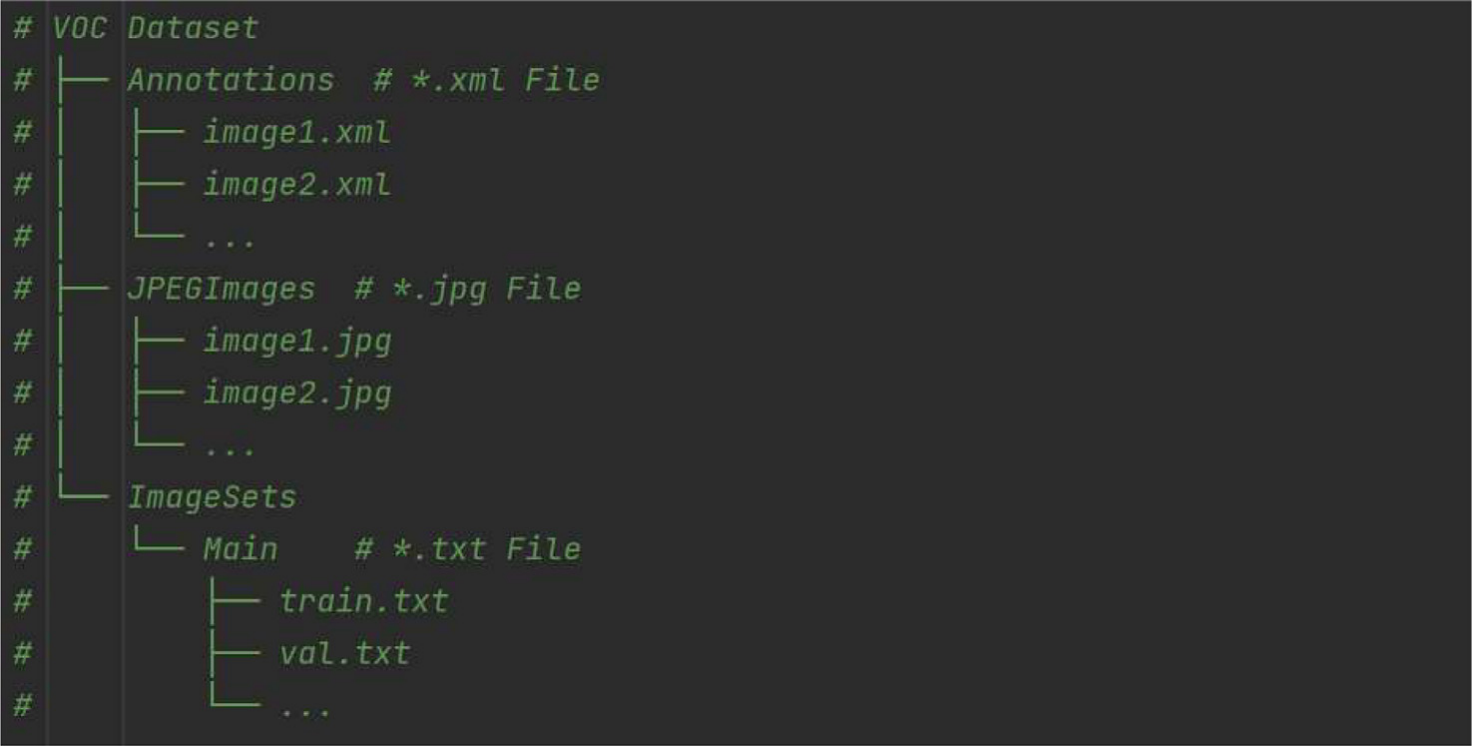
Directory structure of the dataset file.

The criteria for defining different types of pavement distress are as follows: Transverse cracks are herein defined as fissures oriented perpendicularly to the road's central axis or the direction of pavement installation. On the other hand, longitudinal cracks are those that run parallel to the road's central axis or the direction of pavement. Cracks exhibiting angular dispositions within the range of 25 to 70 degrees concerning the road's central axis are categorized as diagonal cracks. The alligator cracks are distinguished by the presence of a sequence of interconnected fissures on the surface of roads. These fissures merge both longitudinally and transversely, resulting in the formation of multifaceted angular fragments, reminiscent of the pattern observed on the back of an alligator. Repairs are indicative of segments of the road surface where fresh materials have been applied for the purpose of replacing and mending the preexisting pavement. Potholes, conversely, are depressions discernible on the road surface, typically assuming a concave, bowl-like shape. They are often characterized by sharp upper edges and vertical sides adjoining the upper rim of the cavity. Sample images of the dataset are shown in Fig. 1, the directory structure of the dataset file is illustrated in Fig. 2.

**Fig. 3:**
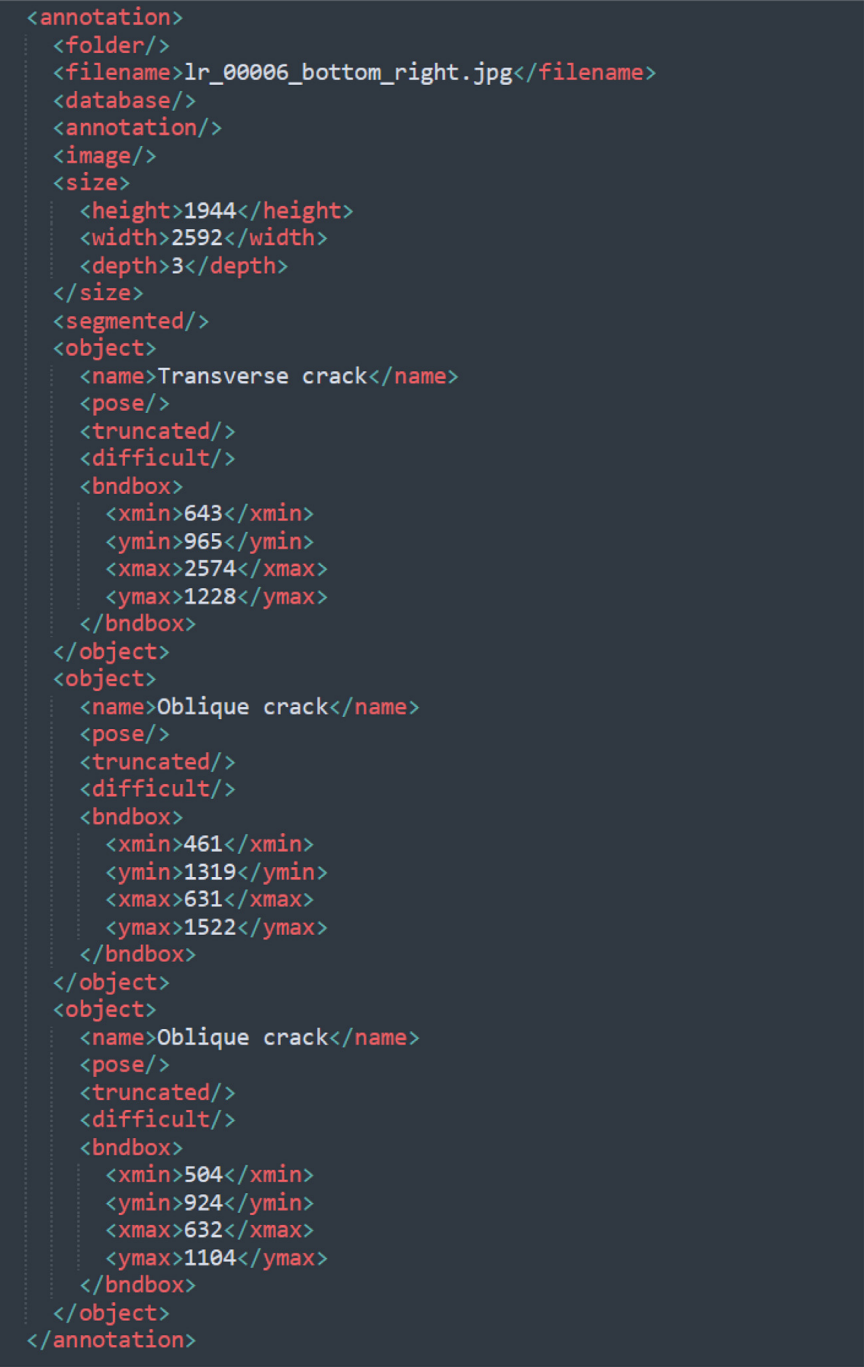
Example of the annotation information in an XML file.

The dataset is divided into three folders: Annotations, ImagesSets and JPEGImages. The Annotations folder contains XML files in PASCAL VOC format [3]. These files include information about the pavement distress types, as well as the coordinates of the bounding boxes. Fig. 3 shows an example of the annotation information in an XML file with one transverse crack and two oblique cracks. The <filename> describes the name of the annotated image, while the <size> indicates the dimensions and number of channels of the image. The<object> denotes the category and position of the bounding boxes. The original images are in the folder JPEGImages.

The file ImageSets contains the names of the images used for training, testing, and validation. The images in the dataset are divided into training, validation, and test sets in appropriate proportions. The ratio of the test set to the trainval set is 2:8, where trainval set refers to the combination of the training and validation sets. Then, the trainval set is further split into the training set and validation set in an 8:2 ratio. This result is provided in four files: “train.txt,” “val.txt,” “test.txt,” and “trainval.txt,” which are located within the “Main” folder inside in the “ImageSets” folder as shown in Fig. 2.

**Fig. 4:**
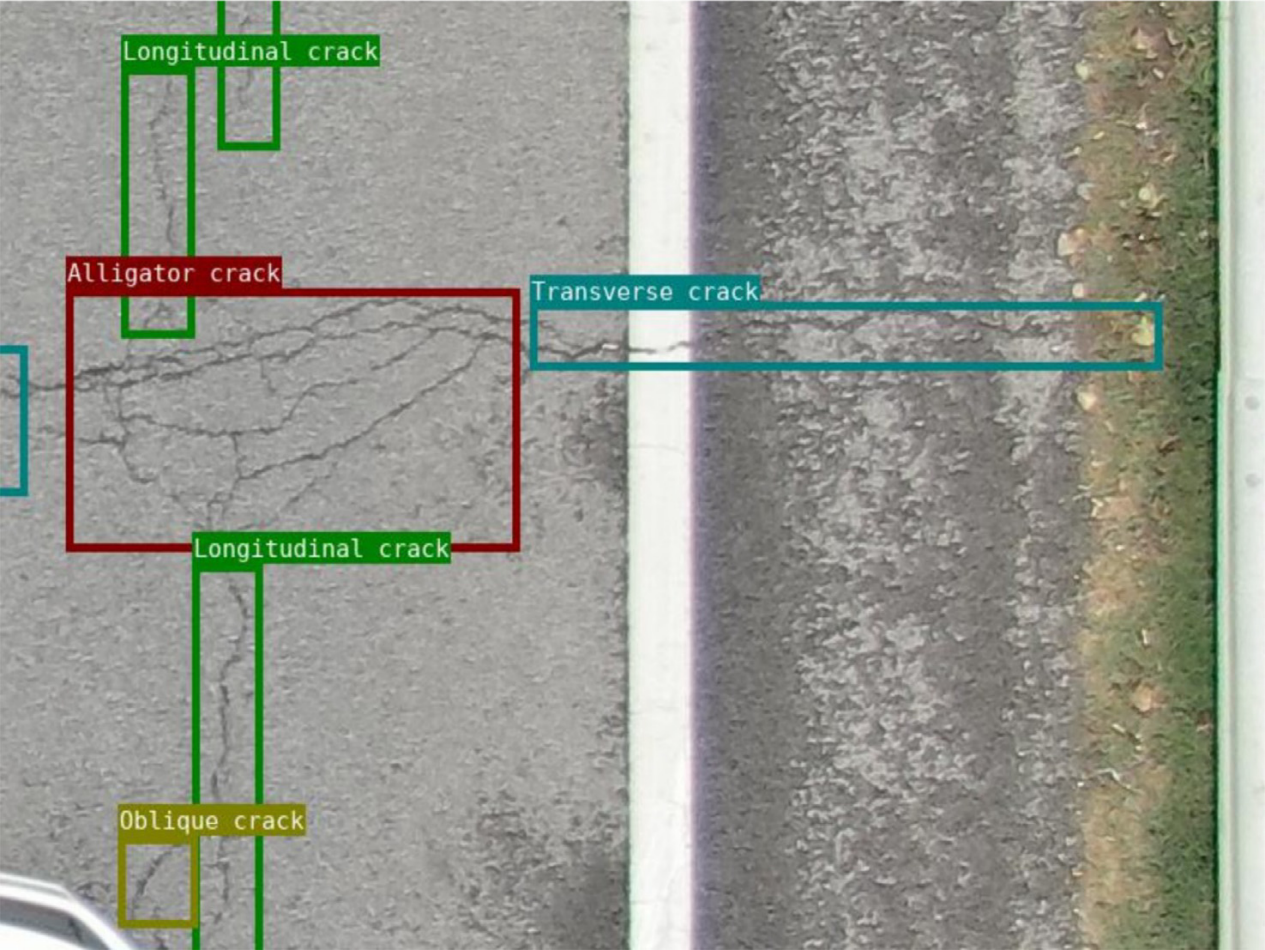
Annotation of the cracks.

We integrated annotation boxes with the images. This approach aids in confirming the accuracy of label position and boundaries, ensuring alignment with actual cracks. Fig. 4 shows the visualization result after the annotation is completed.

The “Images” folder contains 2440 images with a resolution of 2592 × 1944 pixels. The images were taken from different types of roads, including highways, provincial roads, and county roads, satisfying the requirement of pavement distress detection of different types of roads. It is worth noting that the dataset also includes pavement images without distress. These images of well-maintained road are included to facilitate false-positive detection for models developed for pavement distress detection.

## 3 Experimental Design, Materials and Methods

Images were captured using a camera installed on a UAV. we take into account the weather conditions and the quality of road construction when selecting flight routes. We conducted research in multiple locations and captured images from various types of roads.

The scale of a photograph is determined by the focal length of the camera and the flying height above the ground. To select the altitude for UAV flight, the heights of the structures in the shooting area, the size of the roads, and the size of the distress in the images should be considered. Operating at an excessively high altitude may result in the diminishment of pavement distress size in imagery, subsequently exerting an influence on the recognition process. To cover the entire width of the pavement, the minimum flying altitude is set as follows Eq. (1):

(1) $$\frac{f}{H}=\frac{a}{W}$$

where *H* represents the UAV flying altitude; *f* is the focal length of the camera; *a* is the camera sensor size, and *W* is the actual width to be shot. In this study, *a* is 28.2 mm, the focal length *f* is 47mm, and *W* is 18m, so the flight altitude *H* is calculated as 30m. At this height, the crack is still visible and does not affect the recognition.

**Fig. 5:**
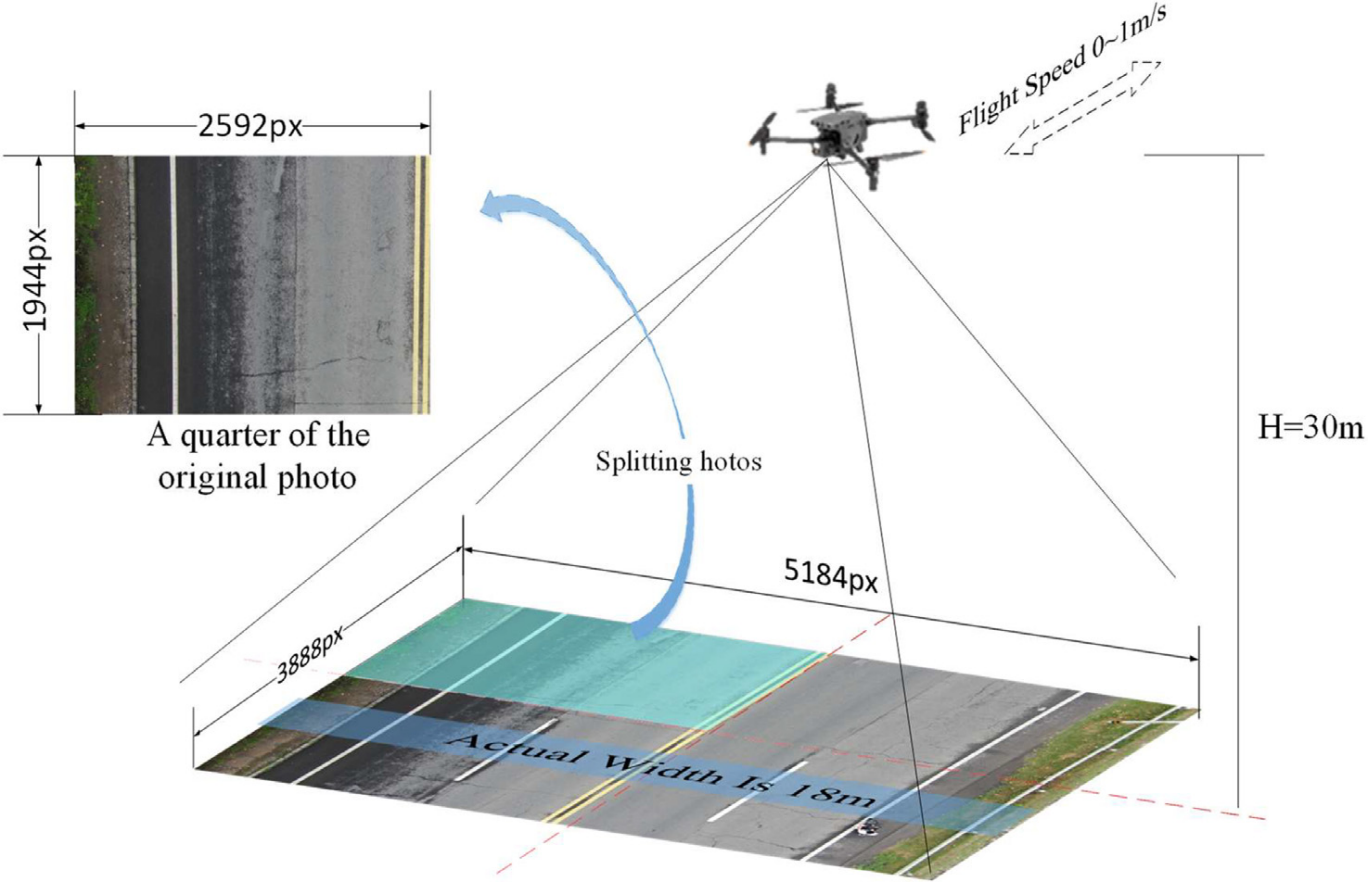
Images captured by UAV.

During the capturing process, the camera of the UAV was positioned vertically downward, perpendicular to the ground. For stationary shooting, the UAV hovered at a fixed point to capture pavement images. For dynamic shooting, the UAV moved at 0.8 m/s to capture pavement images. To diversify the dataset and include different weather conditions, some images were captured one hour after rain. The camera has a resolution of 20 megapixels, with image dimensions of 5184 pixels in width and 3888 pixels in height. The large image size was unsuitable for annotation and algorithm training. Therefore, the images were divided into four equally sized parts: top-left, top-right, bottom-left, and bottom-right, with each individual image having a size of 2592 pixels in width and 1944 pixels in height, as shown in the Fig. 5.

After image screening, image mirroring, and other dataset augmentation techniques, a total of 2440 images were obtained. The cracks were labeled using the *labelme* tool in the PASCAL VOC format [3]. The annotations include different types of distress labels and their locations in the images.

**Fig. 6:**
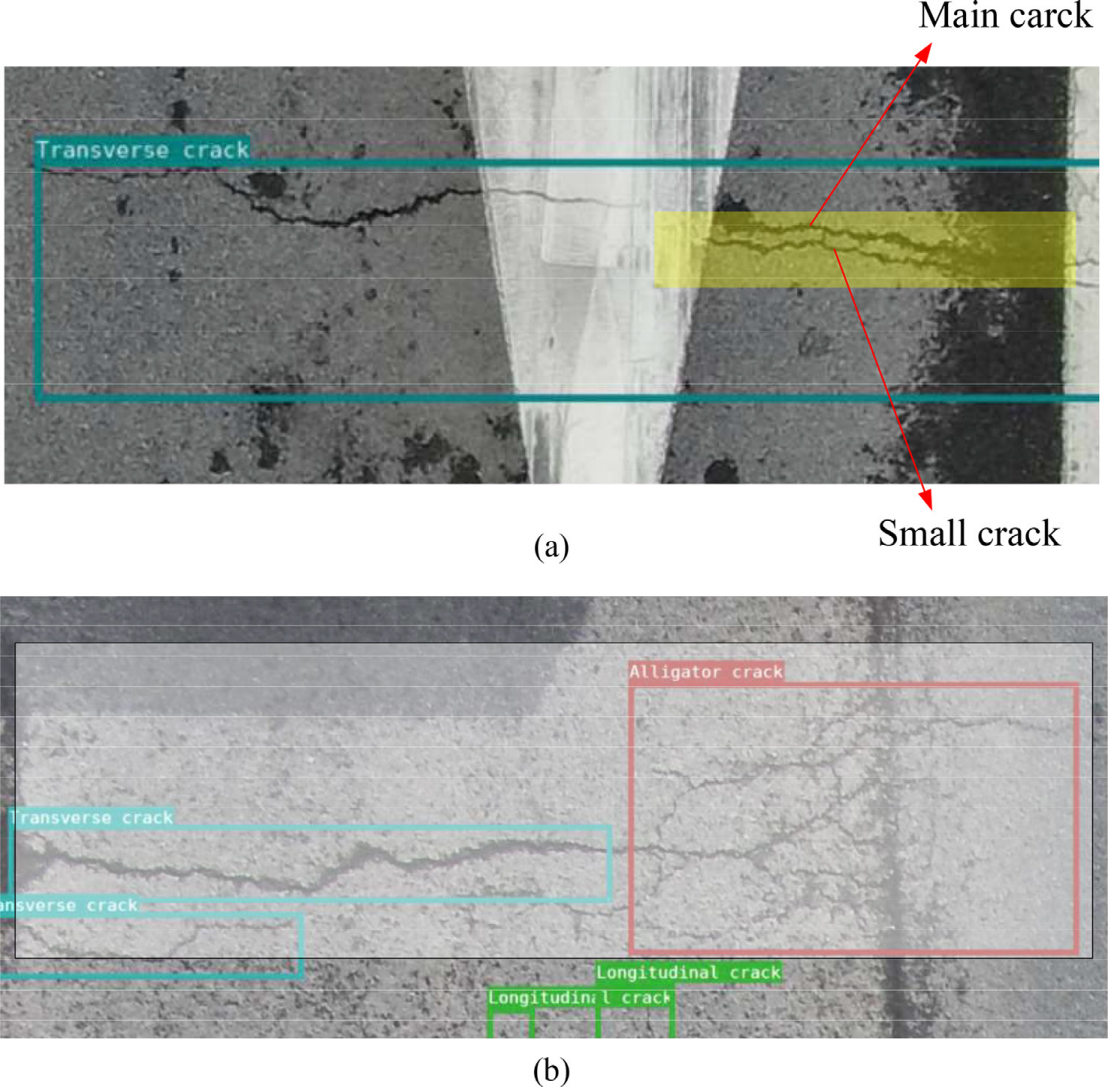
Examples of less distinctive features.

For each annotated image, the image was firstly enlarged ensuring that no cracks were overlooked. In cases where certain target features were less evident, as shown in Fig. 6 (a), there is a short transverse crack in the area covered by the yellow layer, but it is a derivative of the main crack, the two are very close to each other, and the small crack is parallel to the main crack, so the two cracks are labeled as one crack. Fig. 6 (b) in the area covered by the white layer, alligator cracks and a long crack are connected. The cracks are labelled as a transverse crack and an alligator crack.

Six distress types were labeled for the collected images. After completing all the annotation processing tasks, the resulting dataset contains approximately 11,158 annotation boxes.

## 4 Collected Data Analysis

Traditional methods of pavement distress identification, relying on manual observation or pavement inspection vehicles, often involve capturing images from relatively close proximity to the ground [4], resulting in limited image information that hampers model training. Utilizing UAVs for pavement distress detection offers advantages such as cost-effectiveness and environmental friendliness, this approach can effectively reduce road maintenance expenses [5], compared with traditional time-consuming and costly pavement inspection methods.

In this study, a UAV equipped with a high-resolution camera was used to capture pavement distress images to train deep learning, and the best image quality was achieved through reasonable flight settings. Compared with other pavement distress images captured from a relatively close distance with a ground camera [6,7], the images collected by the UAV are taken from a higher altitude and encompass more lanes, significantly improving the efficiency of pavement inspection. Additionally, this study captured images of pavement distresses under two different weather conditions and various road types, enhancing the model's generalization capability. And the dataset categorizes pavement distress into six distinct types and annotates them according to the PASCAL VOC format, simplifying data processing for researchers and enabling developers to focus more on algorithm development. Scholars can utilize this dataset to explore improved algorithm models for UAV image recognition and develop UAV-based pavement inspection systems.

## CRediT authorship contribution statement

**Haohui Yan:** Data curation, Methodology, Writing – original draft. **Junfei Zhang:** Supervision, Writing – review & editing.

## Declaration of Competing Interest

The authors declare that they have no known competing financial interests or personal relationships which have, or could be perceived to have, influenced the work reported in this article.

## Data Availability

UAV-PDD2023 (Original data)
